# Editorial: Tumor Microenvironment: Molecular Mechanisms and Signaling Pathways Involved in Metastatic Progression

**DOI:** 10.3389/fonc.2021.730815

**Published:** 2021-08-13

**Authors:** Wolfgang Link, Gennaro Ilardi, Antonella Zannetti

**Affiliations:** ^1^Instituto de Investigaciones Biomédicas “Alberto Sols” (CSIC-UAM), Madrid, Spain; ^2^Department of Advanced Biomedical Sciences, Pathology Section, School of Medicine and Surgery, University of Naples, Federico II, Naples, Italy; ^3^Institute of Biostructures and Bioimaging, National Research Council (CNR), Naples, Italy

**Keywords:** tumor microenvironment, molecular mechanisms, signal transduction, drug resistance, metastatic process

Despite important advances in cancer treatments, metastasis remains the major cause of cancer mortality. In the complex tumor microenvironment, several malignant and non-malignant cell types, as well as components of the extracellular matrix (ECM), interact together to promote the metastatic process (1, 2). Stromal cells, which are already present in the tumor microenvironment (TME), are recruited from distant sites and “educated” by cancer cells through a dynamic cross-talk involving growth factors, cytokines, chemokines, miRNAs, and exosomes (3, 4). These primed cells, including cancer-associated fibroblasts, tumor-associated mesenchymal stem cells, tumor-associated macrophages, and other immune cells acquire a pro-metastatic function that supports tumor cells in each step of the metastatic cascade (4). Importantly, the bi-directional communication between tumor microenvironment and cancer cells induces the epithelial-mesenchymal-transition (EMT) that in turn elicits profound morphological and functional changes in tumor cells, triggering a mesenchymal-like phenotype with higher invasive potential (5). A crucial step in the establishment of metastases includes the formation of a pre-metastatic niche where recruited stromal cells contribute to creating a favorable microenvironment that permits cancer cell seeding (6). To successfully germ in a distant site, cancer cells need to find nutrients, an ECM that can support their attachment, and stromal cells that help them with paracrine signaling to survive and proliferate in the new environment (1). Responding to this complex scenario, this Research Topic aims to bring together more recent studies and discoveries that shed light on the molecular mechanisms and signaling pathways involved in the cross-talk between TME and cancer cells. The special issue on the **“**Tumor Microenvironment: Molecular Mechanisms and Signaling Pathways Involved in Metastatic Progression” includes 27 original articles, 11 review articles, and three mini-review articles, each of which advances knowledge in this field concerning different types of carcinomas.

## Role of Different Components of the TME in Breast Cancer Metastatic Process

Breast cancer is the most frequently diagnosed cancer and the leading cause of cancer death in women worldwide due to the onset of metastases (7). Here, several articles focus on how the TME impacts breast cancer progression. Our growing understanding of the implications of the TME, in terms of different stromal cell, hypoxic microenvironment, and epigenetic modifications, in the metastatic spread of breast cancer (1) contributes to improving therapeutic approaches to this highly hetereogenous carcinoma in terms of response to treatments, relapse, and metastasis.

The review by D’Esposito et al. addresses the role of the adipose tissue microenvironment in the progression of breast cancer and the involvement of obesity and diabetes in metastasis formation. Obesity is a major risk factor in the development of breast cancer in postmenopausal women. Acheva et al. provide evidence of a possible causal link between these two complex diseases. The authors show that leptin-activated leptin receptors in co-operation with mechanosensitive Ca2+ channels, play a role in the development of breast carcinomas through the regulation of actomyosin dynamics. Liu et al. highlight that the interaction between bone marrow stromal cells and cancer cells plays an important role in breast cancer metastasis to the bone which is the most common metastatic site of this cancer type. Broggi et al. show that the expression of the histone variant macroH2A was higher in metastatic breast cancer compared to non-metastatic cases suggesting this epigenetic regulator as a diagnostic marker. Wu et al. provide evidence for another mechanism underlying hypoxia-related metastasis and resistance to the treatment with paclitaxel in Triple-Negative Breast Cancer through the HIFα-mediated upregulation of Complement 1q Binding Protein (C1QBP). Noted by Gentile et al. in their original research article, depletion of the MAPK kinase kinase MEKK1 in fibroblasts impairs the invasion of breast tumor cells, an effect shown to be associated with reduced basal and tumor cell-induced expression of the chemokine CCL5.

Another original research study authored by Xiong et al. investigates the mechanism by which depletion of NOTCH3 interferes with mammary gland development in mice. RNA Sequencing revealed that the expression of chemokine ligand 2 (CCL2) was reduced in mammary gland tissues from mice with heterozygous or homozygous germline deletion of Notch3. Notch3-dependent CCL2 expression is postulated as an important event in mammary gland development and as a target for breast cancer therapy.

## Epithelial-Mesenchymal Transition (EMT) and Cancer Stem Cells (CSCs) as Drivers of Metastasis

EMT is a basic developmental process that converts epithelial cells to mesenchymal cells. Although the correlation of EMT with cancer metastasis is well-known, the molecular mechanisms for it remain to be fully clarified. There is a strong relationship between EMT and CSCs in promoting cancer progression as well as inducing drug resistance and metastases (8, 9). In this Research Topic, a number of authors address this issue and elucidate the roles of EMT and CSC in the aggressiveness of several tumors. The phosphatidylinositol 3−kinase (PI3K)/protein kinase B (AKT) and Wnt/β-catenin are reported as the main signaling pathway involved in the regulation of EMT and CSCs.

Wang et al. show that the expression of the Kinesin Family Member 3B (KIF3B) was up-regulated in breast cancer tissues and cell lines, while its knockdown suppressed tumor cell proliferation and migration. The authors conclude that the inhibition of KIF3B suppresses the Wnt/β-catenin signaling pathway and EMT in breast cancer. The original research study authored by Kiran et al. reports another mechanism involved in EMT. The authors provide evidence that overexpression of the NAD+ dependent deacetylase sirtuins 7 (SIRT7) in mesenchymal cells induces EMT accompanied by N- to E- cadherin transition, stabilization of β-catenin, and the downregulation of Snail, Slug, and Zeb1, transcription factors responsible for maintenance of mesenchymal phenotype. Tumor-associated hypoxia and in particular hypoxia-inducible factor-1α (HIF-1α) signaling have been linked to tumor aggressiveness, EMT, and metastasis. The review article by Tam et al. summarizes recent reports on hypoxia-induced EMT by HIF-1α or other potential mediators. Similarly, aberrant PI3K/AKT signaling is considered one of the most frequent events in human tumors and is known to be involved in key features of cancer aggressiveness. Park et al. contribute an original research article providing evidence that PI3K/AKT signaling may be linked to multiple CSC characteristics in colorectal cancer, such as radio-resistance, stem-like property, and tumorigenic potential. The study shows that caffeic acid effectively targets colorectal CSC populations by inhibiting the growth and/or self-renewal capacity of colorectal CSCs through PI3K/AKT signaling *in vitro* and *in vivo*. A central hub in this signaling pathway is the serine/threonine kinase AKT that regulates the activity of other signaling proteins by phosphorylation. One member of the ever-growing list of AKT substrates is β-catenin. Lee et al. show in their study that the rate-limiting, glycolytic enzyme, phosphofructokinase 1 platelet isoform (PFKP) promotes epidermal growth factor receptor (EGFR) activation and subsequent AKT-mediated β-catenin phosphorylation. This event leads to the nuclear translocation and activation of β-catenin, thereby enhancing the expression of its downstream genes CCND1 and MYC in human glioblastoma cells. Similarly, Liu et al. establish a link between the Nuclear factor-κB activating protein (NKAP) and the PI3K/AKT pathway. NKAP acts as an oncoprotein in various cancers and is associated with a poor prognosis. The authors find NKAP overexpressed in recurrent neuroblastomas (NBs) when compared with non-recurrent NBs and that NKAP silencing attenuated PI3K/AKT signaling inhibiting proliferation and promoting apoptosis. The cytosolic tyrosine phosphatase Shp1 has emerged as a tumor suppressor in several cancer types. In their mini-review, Varone et al. highlight that the tumor suppressor activity of Shp1 is due to its ability to attenuate or terminate signaling pathways controlling cell proliferation, survival, migration, and invasion.

## TME Involvement in Malignant Evolution of Gastrointestinal Carcinomas

Gastrointestinal cancers represent a heterogeneous complex class that is characterized by a critical interplay of genetic and environmental factors that drive the conversion of normal tissue to premalignant lesions, and finally to malignancy (10). Two interesting reviews elucidate the crucial role played by colorectal CSCs in promoting metastasis, particularly to the liver, by discussing the main signal pathways involved, their ability to escape immunosurveillance, and innovative therapeutic approaches targeting CSCs (Lin et al.; Gonzalez-Villareal et al.). CD44 is a well-known CSC marker and the variant CD44v9 has been closely associated with cell proliferation, metastasis, and tumor invasiveness through EMT in human cholangiocarcinoma (Suwannakul et al.). Similarly, Martincuks et al. report an in-depth examination of the role of CD44 in ovarian cancer and the importance of functional crosstalk between CD44 and STAT3. Li et al. report that in colorectal cancer cells and animal models, miR-1224-5p represses the EMT program, migration, and invasion exerting its function by directly targeting the SP1-mediated NF-kB pathway.

A comprehensive analysis of the diverse roles of mucin genes in the diagnosis, treatment, and prognostic evaluation of patients with colorectal cancer (CRC) is also discussed in this special issue (Gan et al.). The receptor for advanced glycation end-products (RAGE) pathway is strongly related to the abundant malignant behavior of CRC cells, including chemoresistance, invasion, and proliferation. These findings propose RAGE as a crucial player in the connection between inflammation and colon carcinogenesis (Azizian-Farsani et al.). Luo et al. report that an immune prognostic model (IPM) could identify subgroups of metastatic CRC (mCRC) with different recurrence risks and stratify the mCRC samples sensitive to Immuno-/chemotherapy with biologically explainable evidence. Furthermore, they also highlight the importance of MHC class-II molecules in immunotherapy of mCRC.

Esophageal squamous cell carcinoma (ESCC) is another group of gastrointestinal carcinomas with high metastatic potential. Fu et al. demonstrate that the WNT2 ligand could stabilize and phosphorylate the FZD2 receptor by attenuating FZD2 ubiquitination, leading to the activation of STAT3 signaling and the initiation of esophageal cancer cell metastasis. Maternal embryonic leucine zipper Kinase, MELK, enhances tumorigenesis, migration, invasion, and metastasis of ESCC cells *via* activation of the FOXM1 signaling pathway, suggesting MELK is a potential therapeutic target for ESCC patients, even those in an advanced stage (Chen L et al.). Through lipidomic methods, Li et al. identify the specific lipid metabolites in tumor tissues and blood plasma samples of patients with ESCC and evaluate the effects of angustoline on viability, migration, and invasion of cancer cells as well as the impact of angustoline on the LKB1/AMPK/ELAVL1/LPCAT2 pathway. Based on the results of the circRNA array on ESCC plasma samples, Wang et al. first identify a circular RNA generated from the ZDHHC5 gene, which is termed circ-ZDHHC5, and elucidate how it could impact ESCC progression by sponging miR-217 with ZEB1, an EMT associated transcription factor. In their review, Chen et al. describe the main mechanisms underlying the ability of gastrointestinal cancer to metastasize to ovarian tissue thus giving rise to metastatic ovarian cancer (MOC). Lei et al. demonstrate that the number of Nanog-positive cells positively correlates with poor prognosis in patients with hepatocellular carcinoma (HCC); whereas another study performed by Liu et al. shows that Secreted Protein Acidic And Cysteine Rich (SPARC) can facilitate proliferation and metastasis of HCC *via* modulation of the ERK1/2-MMP2/9 signaling pathway.

## Contribution of the TME to the Progression of Other Tumor Types

Lung cancer and osteosarcoma are carcinomas with high metastatic potential. Many studies are therefore focusing on examining the molecular mechanism underlying the invasiveness of tumor cells. Zhu et al. report an overview of current research on lung cancer metastasis, including molecular pathways, anatomical features, and the genetic traits that make lung cancer intrinsically metastatic. The proliferation-inhibiting role of calcium sensing receptor in lung adenocarcinomas is demonstrated by Li’s group as well as the involvement of GSK3b/Cyclin D1 pathway (Li et al.). Ren et al. show that targeting the miR-1260b/SFRP1/Wnt signaling axis might provide a novel strategy for overcoming chemotherapy resistance in lung adenocarcinoma. An epigenetic change in the promoter region of miR-199a contributes to the aggressive behavior of papillary thyroid carcinoma *via* the miR-199a-3p/DNMT3a regulatory circuit and directly targets RAP2a (Wu et al.).

The findings by Luo et al. highlight small extracellular vesicles packaging of miR-19a-3p as a potential target for prevention and treatment of bone destruction and cancer progression in osteosarcoma patients. Interestingly, Zheng et al. report that the vascular endothelial growth factor receptor-2 (VEGFR2) can affect both osteosarcoma cell metastasis and immune escape through the deactivation of STAT3 and the RhoA-ROCK-LIMK2 pathway. High expression of DDX19A is positively correlated with metastasis and poor clinical outcome in cervical squamous cell carcinoma (Jiang et al.). A gain of function mutation in MyD88 (MyD88 L265P) enhances the NF-kB and JAK-STAT signaling pathways and is associated with dysregulation of Toll-like receptor (TLR) signaling in the pathogenesis of Diffuse large B cell lymphoma (An et al.). Broggi et al. show in uveal melanoma that high levels of Beclin-1 correlate with a lower risk of metastasis and higher disease-free survival times, indicating a positive prognostic role for Beclin-1. Santagata et al. focus on the crucial role played by CXCR4 and CXCR7 in promoting tumor progression and immune-stromal cell recruitment in multiple human cancers. Arora and Pal shed light on the diversity of stromal and immune cell populations, whose subtypes create the complexity of TME, leading primary tumors towards advanced stage cancers. Many studies reviewed by Wang et al. show that nerves infiltrate the TME enhancing cancer growth and metastasis and perineural invasion is a process by which cancer cells invade the surrounding nerves.

Taken together, the articles published in this Research Topic provide an overview of the complex interplay of cellular and acellular components such as malignant and non-malignant cell types, components of the ECM, and the signaling cascades and secreted factors involved that are capable of driving metastasis in different tumor types, in particular in breast and gastrointestinal cancers. This special issue collects original research and review articles on recent achievements in understanding TME biology with particular attention to the crucial role played by the EMT program and niche of CSCs in contributing to the metastatic process and on the development of therapeutic strategies to target these processes for improving the clinical outcome of anticancer therapies.

## Author Contributions

WL, GI, and AZ wrote, reviewed, and edited the manuscript. All authors have read and agreed to the published version of the manuscript.

## Funding

This work was supported by the Spanish Ministry of Science, Innovation, and Universities through Grant RTI2018-094629-B-I00 to WL. This work was supported by grants from from MIUR Progetti di Ricerca di Rilevante Interesse Nazionale (PRIN) Bando 2017-grant 2017MHJJ55, MIUR-PON “Ricerca e Innovazione” 2014-2020 (grant MOLIMONCOBRAIN LAB), from Regione Campania PO FESR 2014-2020 (grant eMORFORAD) to AZ.

## Conflict of Interest

WL is the scientific co-founder of Refoxy Pharmaceuticals GmbH, Berlin, and is required by his institution to state so in his publications. The funders had no role in the design and writing of the manuscript.

The remaining authors declare that the research was conducted in the absence of any commercial or financial relationships that could be construed as a potential conflict of interest.

## Publisher’s Note

All claims expressed in this article are solely those of the authors and do not necessarily represent those of their affiliated organizations, or those of the publisher, the editors and the reviewers. Any product that may be evaluated in this article, or claim that may be made by its manufacturer, is not guaranteed or endorsed by the publisher.
